# Astragaloside IV: a potential nemesis for gastric cancer

**DOI:** 10.3389/fphar.2025.1636341

**Published:** 2025-07-18

**Authors:** Chao Hu, Qiong Li, Song-Nan Gong, Xiao-Jie Zou, Jia-Yue Xu, Hai-Feng Ying, Lan Zheng

**Affiliations:** ^1^ Department of Traditional Chinese Medicine, Loujiang New City Hospital of Taicang (Taicang Branch of Ruijin Hospital Affiliated with Shanghai Jiao Tong University School of Medicine), Suzhou, China; ^2^ Department of Hepatology, Yueyang Hospital of Integrated Traditional Chinese and Westen Medicine, Shanghai University of Traditional Chinese Medicine, Shanghai, China; ^3^ Department of Gastroenterology, Loujiang New City Hospital of Taicang (Taicang Branch of Ruijin Hospital Affiliated with Shanghai Jiao Tong University School of Medicine), Suzhou, China; ^4^ Department of Public Health, Loujiang New City Hospital of Taicang (Taicang Branch of Ruijin Hospital Affiliated with Shanghai Jiao Tong University School of Medicine), Suzhou, China; ^5^ Department of Traditional Chinese Medicine, Ruijin Hospital, Shanghai Jiao Tong University School of Medicine, Shanghai, China

**Keywords:** gastric cancer, astragaloside IV, apoptosis, mechanisms, review

## Abstract

Gastric cancer (GC), a life-threatening malignancy with profound global health impacts, remains a cardinal focus of biomedical research. Recently, astragaloside IV (AS-IV), a bioactive triterpenoid saponin derived from *Astragalus mongholicus Bunge*, has garnered substantial attention for its multifaceted anticancer properties in preclinical investigations. This review systematically synthesizes current evidence on the molecular mechanisms underlying AS-IV’s inhibitory effects against GC, encompassing programmed cell death pathways (apoptosis, autophagy, pyroptosis, ferroptosis), tumor angiogenesis, tumor microenvironment modulation, *Helicobacter pylori* and inflammatory signaling networks. Many studies demonstrate that AS-IV can inhibit the development of GC through multi-target and multi-pathway mechanisms, making it a well-deserved nemesis of GC. Notably, although AS-IV has emerged as a potential candidate for GC therapy, it suffers from problems such as single research model, unclear toxic and side effects, and poor bioavailability. These seriously hinder the efficiency of AS-IV in the treatment of GC. In the future, we can design and implement a series of *in vivo* and *in vitro* experiments to further explore and clarify the mechanism of action of AS-IV in the treatment of GC. It is encouraged to carry out a number of high-quality clinical controlled studies to further prove the effectiveness and safety of AS-IV. In addition, we can also use emerging technologies (such as nanotechnology) to improve the bioavailability of AS-IV, bringing more hope to GC patients.

## 1 Introduction

Gastric cancer (GC) a highly aggressive malignancy originating from gastric mucosal epithelial cells, is characterized by alarmingly high incidence and mortality rates globally. Data from the International Agency for Research on Cancer (IARC) reveal that in 2022 alone, the world witnessed 970,000 newly diagnosed GC cases and 660,000 GC-related fatalities (International Agency for Research on Cancer, 2024). The upward trend in both the incidence and mortality of GC persists unabated, particularly pronounced in East Asian countries, including China, Japan, and South Korea (Li M. et al., 2025). This persistent increase underscores the urgent need for more effective prevention and treatment strategies, as GC continues to pose a significant threat to human health. Although remarkable advancements have been made in the treatment of GC, encompassing surgical resection, chemotherapy, targeted therapy, immunotherapy, and radiotherapy, these modalities are not without limitations (Smyth et al., 2020). Despite the tangible improvements in patient outcomes, challenges such as drug resistance, severe adverse effects, and high recurrence rates still hinder the achievement of optimal treatment results. These limitations highlight the necessity of exploring novel therapeutic approaches to further improve the prognosis of GC patients.

The etiology and pathogenesis of GC remain unclear. Current evidence suggests that gastric carcinogenesis is intricately influenced by both endogenous factors, primarily genetic predisposition, and exogenous factors, predominantly environmental exposures (Shah and Bentrem, 2024). Among these, *Helicobacter pylori* (*H. pylori*) infection has emerged as a pivotal exogenous determinant in GC initiation and progression, with a particularly high prevalence in regions such as East Asia and Africa (Li et al., 2023). *Helicobacter pylori*, a gram-negative *bacillus*, is commonly detected in patients with chronic gastritis, and initially discovered by Warren JR and Marshall B in 1982 (Warren and Marshall, 1983). More research has revealed that *H. pylori* employs a multi-faceted mechanism to drive gastric carcinogenesis. By secreting virulence factors such as cytotoxin-associated gene A (CagA) and vacuolating cytotoxin A (VacA), *H. pylori* activates multiple oncogenic signaling pathways, including the mitogen-activated protein kinase (MAPK) and phosphatidylinositol 3-kinase/protein kinase B (PI3K/Akt) pathways. This activation leads to the disruption of normal gastric epithelial cell function, triggering abnormal apoptosis and proliferation, damaging the gastric mucosa, and initiating a cascade of pathological events, including chronic inflammation, intestinal metaplasia, and dysplasia, ultimately culminating in GC (Shah and Bentrem, 2022). Additionally, *H. pylori*-induced production of reactive oxygen species (ROS) and reactive nitrogen species (RNS) causes DNA damage, which, in turn, promotes oncogene activation, tumor suppressor gene inactivation, and epigenetic alterations. These genetic and epigenetic changes modify the host’s genetic susceptibility and reshape the gastric microenvironment, further facilitating tumorigenesis (Salvatori et al., 2023). Epidemiological studies have estimated that *H. pylori* infection contributes to 89% of non-cardia GCs and 18% of cardia GCs (Plummer et al., 2016). It is crucial to note that *H. pylori* infection is merely one of the many etiological factors involved in GC development. Other contributing factors include age, genetic inheritance (Jin et al., 2020), psychological stress (Kouhestani et al., 2022), smoking (Rota et al., 2024), alcohol consumption (Deng et al., 2021), high-salt diet (Wu et al., 2022), as well as socioeconomic status, personal hygiene habits, and the overall level of social development (Zhang R. et al., 2021). Collectively, these factors interact in a complex manner, underscoring the multifactorial nature of gastric carcinogenesis and the necessity for a comprehensive approach to prevention and intervention.

More specifically, the pathogenesis of GC results from the combined action of multiple factors. In addition to *H. pylori*, dietary nitrites can be converted into carcinogenic nitrosamines within the stomach, and long-term excessive intake thereof increases the risk of cancer. Epstein-Barr virus (EBV) infection induces abnormal proliferation of gastric mucosal cells by regulating host gene expression. Harmful substances from alcohol and smoking damage the gastric mucosa, disrupt its barrier function, activate inflammatory responses, and promote tumorigenesis. Furthermore, genetic mutations are also critical contributors—for example, Cadherin 1 (CDH1) gene mutations in diffuse GC reduce intercellular adhesion, enabling tumor cells to invade and metastasize more readily. These factors either directly damage cellular DNA or indirectly promote tumor progression through chronic inflammation, metabolic disorders, etc., acting synergistically to drive the initiation and development of GC.

In recent years, there has been a surge of interest in monomeric metabolites derived from traditional Chinese medicine (TCM) due to their promising anticancer activities through multi-targeted and multi-pathway mechanisms, with an exponentially growing body of research dedicated to this field (Wen et al., 2024). Astragaloside IV, the principal bioactive metabolite of *Astragalus mongholicus Bunge* and a pivotal material basis for its pharmacological efficacy, is categorized as a tetracyclic triterpenoid saponin (Stępnik et al., 2025). Cumulative evidence from preclinical and translational studies has established AS-IV as a potential anticancer agent with broad-spectrum antitumor activity. Through synergistic modulation of diverse molecular pathways, AS-IV exerts pronounced inhibitory effects against a wide array of malignancies, including GC (Chen et al., 2023), colorectal cancer (Zhou et al., 2024), lung cancer (Wu et al., 2024), nasopharyngeal carcinoma (Zeng et al., 2025), breast cancer (Jiang et al., 2017), cervical cancer (Shen et al., 2023), renal cell carcinoma (Wang et al., 2025), hepatocellular carcinoma (Fang et al., 2023), and prostate cancer (You et al., 2023). Although some studies in recent years have clarified the anti-cancer mechanism of *Astragalus mongholicus Bunge*, they have not deeply explored its core components. For example, studies have shown that *Astragalus mongholicus Bunge* inhibits GC through multiple mechanisms, but it is unclear which component dominates this mechanism (Tibenda et al., 2024). Additionally, some studies suggest that AS-IV can inhibit various tumors through multiple programmed cell death pathways, yet none specifically focus on GC (Sheng et al., 2024). This paper synthesizes the advantages and disadvantages of the above-mentioned studies, and for the first time systematically expounds that AS-IV—the core component of *Astragalus mongholicus Bunge*—treats GC through multiple pathways, involving both programmed cell death mechanisms and the relatively novel ferroptosis theory.

This review aims to provide a comprehensive and systematic overview of the current understanding of AS-IV’s molecular mechanisms in GC, thereby offering a robust theoretical framework and evidence-based strategies for its development as a therapeutic agent in gastric oncology. The literature retrieval methods are as follows. Our literature screening process was conducted in PubMed and Web of Science databases. The key search terms included “gastric cancer”, “Astragaloside Ⅳ”, and “mechanism”, with expanded terms such as “traditional Chinese medicine”, “proliferation”, “apoptosis”, “autophagy”, “pyroptosis”, “tumor angiogenesis”, “ferroptosis”, “tumor microenvironment”, “inflammation”, and “*Helicobacter pylori*”. Logical operators applied included “AND” (intersection), “OR” (union), and “NOT” (exclusion). And the literature is required to be published within the last 5 years. We have appropriately relaxed the publication timeline for certain literatures due to their traceability and classic perspectives. In addition, we will have dedicated personnel to eliminate some literatures that do not meet the standards, such as review articles, case reports, experience summaries, monographs, and literatures with low-quality evidence, etc.

## 2 Sources of AS-IV

**FIGURE 1:**
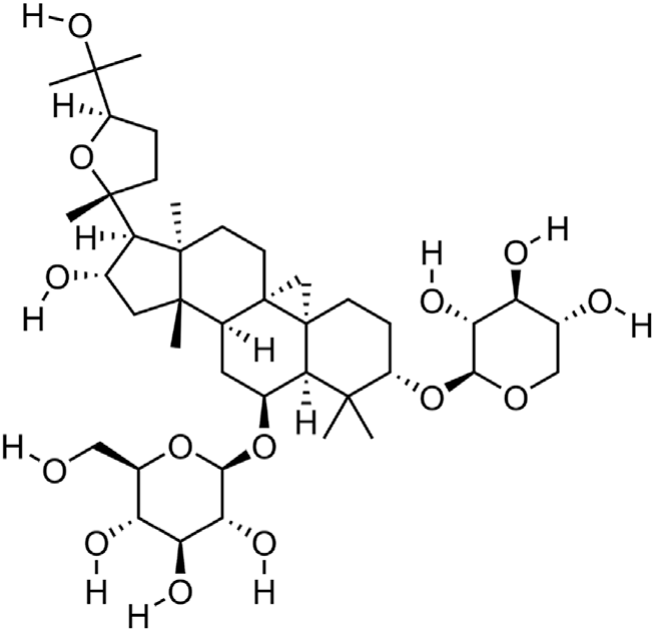
Molecular structure diagram of AS-IV,From National Center for Biotechnology Information (2025). PubChem Compound Summary for CID 13943297, Astragaloside IV. Retrieved 27 May 2025 from https://pubchem.ncbi.nlm.nih.gov/compound/Astragaloside-IV.

Astragaloside IV, a naturally occurring tetracyclic triterpenoid saponin, is composed of an aglycone moiety and glycosyl chains, with a defined molecular formula of C_41_H_68_O_14_ (41 carbon atoms, 68 hydrogen atoms, and 14 oxygen atoms). This unique structural architecture confers upon it a broad spectrum of biological activities (Chen et al., 2025) (Figure 1). Contemporary pharmacological investigations have revealed that AS-IV exerts multifaceted effects, including anti-inflammatory (Li H. L. et al., 2024), antioxidant (Zhang J. et al., 2021), anti-apoptotic (Su et al., 2020), and immunomodulatory properties (Song et al., 2014). Astragaloside IV is derived from *Astragalus mongholicus Bunge* and serves as its core component. *Astragalus mongholicus Bunge* belongs to the genus *Astragalus* in the legume family (Fabaceae), and is a perennial botanical drug. Its fleshy and thick roots are the main medicinal part. Wild populations are mostly distributed from Siberia to the Russian Far East, as well as the western and northern regions of China. As a perennial plant, it mainly grows in temperate biomes (Fu et al., 2014). As the principal bioactive metabolite of *Astragalus mongholicus Bunge*, AS-IV is typically isolated through a standardized extraction protocol: solvent-based extraction using ethanol followed by purification via chromatographic techniques, such as macroporous resin adsorption and silica gel column chromatography.

## 3 Metabolic pathways of AS-IV

The absorption and metabolism of AS-IV predominantly occur in the intestinal tract, liver, and kidneys (Cheng and Wei, 2014). Following oral administration, intestinal microbiota-mediated hydrolysis of the glycosidic bond in AS-IV generates astragenin, its aglycone metabolite. This process enhances the lipophilicity of astragenin, thereby facilitating its transintestinal absorption into the systemic circulation. Once absorbed, AS-IV and its metabolites distribute to multiple tissues, including the liver, kidney, spleen, and lung, albeit at low concentrations in both blood and tissues. The liver, as the primary metabolic organ, exhibits relatively higher drug accumulation. Hepatic metabolism involves cytochrome P450 (CYP450)-mediated oxidation and hydrolysis, followed by biliary excretion into the intestinal lumen. In the gut, β-glucuronidase produced by commensal microbiota hydrolyzes the metabolites, releasing free AS-IV or astragenin for reabsorption and subsequent reincorporation into the bloodstream, thus establishing an enterohepatic circulation (Takeuchi et al., 2022). Final elimination primarily occurs via renal excretion, with minimal elimination of the unchanged parent metabolite (Du et al., 2005).

In contrast to oral administration, AS-IV is rapidly absorbed and widely distributed in various tissues of the body after intravenous injection. It achieves the highest concentrations in the liver and kidneys, followed by the lungs and heart. In Beagle dogs, when AS-IV was intravenously injected at doses of 0.5, 1, and 2 mg/kg, the half-lives were 177.18, 196.58, and 241.59 min respectively, and the total body clearances were 4, 4, and 3 mL/kg/min in sequence. It can be seen that with the increase of dose, the half-life is prolonged and the clearance rate is slightly decreased, suggesting that the drug elimination may slow down at high doses. When administered orally at 10 mg/kg, the half-life was 229.71 min and the clearance rate was 10 mL/kg/min, which was higher than that of intravenous injection, possibly related to the first-pass effect of oral administration. Overall, it shows that the dose is positively correlated with the half-life and weakly negatively correlated with the clearance rate, and the administration route affects the clearance rate (Zhang et al., 2007). Regrettably, relevant research remains limited, and there is an urgent need for more animal and human experiments in the future to clarify the pharmacokinetics of AS-IV.

## 4 Mechanisms of AS-IV in the treatment of GC

### 4.1 Proliferation

Cell proliferation constitutes the fundamental basis for tumor initiation and progression (Li P. et al., 2024). The cell cycle, an orchestrated sequence of events governing cell growth and division, is composed of the G1, S, G2, and M phases, each regulated by stringent molecular checkpoints that govern phase transitions. Dysregulation of these regulatory mechanisms can culminate in uncontrolled cellular proliferation, a hallmark event in tumorigenesis (Gao et al., 2018). Consequently, inhibiting the aberrant proliferation of GC cells represents a critical therapeutic strategy in GC.

In a seminal study by Li F. and colleagues, AS-IV was shown to suppress GC cell proliferation and metastatic potential through downregulation of circular RNA dihydrolipoamide S-succinyltransferase (circDLST) (Li F. et al., 2022). Previous studies have established that circDLST promotes GC progression (Zhang et al., 2020). Mechanistically, circDLST acts as a “sponge” for microRNA-489-3p (miR-489-3p), sequestering the micro Ribonucleic Acid (microRNA) and relieving its inhibition of Eukaryotic Translation Initiation Factor 4A1, (EIF4A1,a direct target of miR-489-3p). AS-IV disrupts this axis by reducing circDLST expression, thereby releasing miR-489-3p from circDLST-mediated sequestration. Elevated miR-489-3p levels enhance the suppression of EIF4A1, ultimately inhibiting GC cell proliferation and metastasis.

Notably, this study represents the first to delineate the circDLST/miR-489-3p/EIF4A1 signaling axis as a key mediator of AS-IV’s antitumor activity in GC, providing a robust mechanistic framework for the development of AS-IV as a novel therapeutic agent in GC treatment. These findings underscore the potential of AS-IV to target non-coding RNA networks for cancer therapy, offering new insights into the molecular mechanisms underlying TCM-derived metabolite efficacy. However, it must be pointed out that current research on AS-IV inhibiting GC cell proliferation is extremely limited. This study lacks sufficient corroboration, and whether there are other underlying mechanisms still requires further investigation.

### 4.2 Apoptosis

Inducing apoptosis represents a critical therapeutic mechanism for GC treatment. Apoptosis, a genetically regulated program of programmed cell death, is fundamental to maintaining normal physiological balance and internal environmental stability in the body (D'Arcy, 2019). During the development of tumor, apoptotic mechanisms are often suppressed, leading to abnormal proliferation and survival of cancer cells (Kashyap et al., 2021).

Xia D. et al. demonstrated that AS-IV induces apoptosis in GC cells via the mitochondrial pathway. Mechanistically, AS-IV exerts direct effects on AGS cells, leading to decline of mitochondrial membrane potential, increased mitochondrial membrane permeability, and subsequent release of cytochrome C from mitochondria into the cytosol. This initiates a cascade of events culminating in the activation of caspase family proteases (Caspase-3, Caspase-7, and Caspase-9) via proteolytic cleavage, ultimately driving apoptotic cell death (Xia et al., 2023). Notably, caspase-3 serves as a central executor of mitochondrial apoptosis in this context (Eskandari and Eaves, 2022).

**FIGURE 2:**
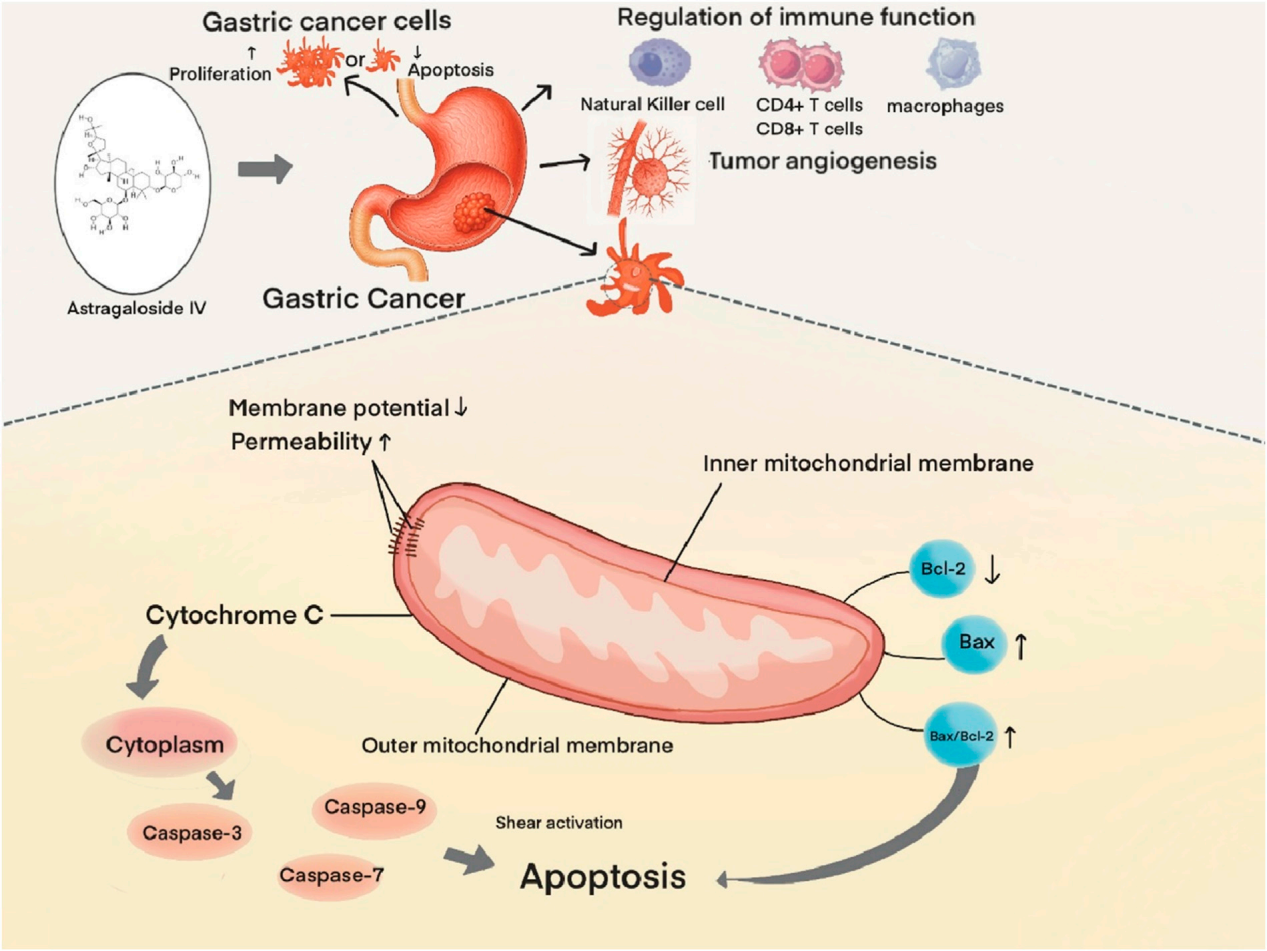
AS-IV induces apoptosis of gastric cancer cells through the mitochondrial pathway.

AS-IV further modulates the mitochondrial pathway through regulation of B-cell lymphoma 2 (Bcl-2) family proteins, which comprise anti-apoptotic (e.g., Bcl-2, B-cell lymphoma-extra large [Bcl-xL])and pro-apoptotic (e.g., Bcl-2 associated X protein [Bax], Bcl-2 homologous antagonist/killer [Bak]) members that play pivotal roles in apoptotic regulation (Campbell and Tait, 2018). Experimental evidence demonstrates that AS-IV upregulates Bax expression while downregulating Bcl-2, resulting in an elevated Bax/Bcl-2 ratio. This shift in protein balance promotes mitochondrial outer membrane permeabilization (MOMP) and subsequent release of apoptotic factors, thereby amplifying the apoptotic response (Zhao et al., 2019) (Figure 2).

There is a close mechanistic connection between cell proliferation and apoptosis, and the two together constitute an important pathway for its anti-GC effect. For example, proliferation inhibition creates conditions for apoptosis induction, while apoptosis induction strengthens the effect of proliferation inhibition. This is specifically manifested in the formation of a synergistic network through mechanisms such as cell cycle regulation, cross-talk of signaling pathways, and mitochondrial dysfunction. This dual action mode of “inhibition-clearance” demonstrates unique advantages in anti-GC research, providing a theoretical basis for the development of anti-tumor strategies based on AS-IV.

### 4.3 Autophagy

Autophagy, a conserved programmed catabolic process, enables cells to degrade intracellular components—including damaged organelles, misfolded proteins, and intracellular pathogens—via lysosomal machinery, serving as a fundamental mechanism for maintaining cellular homeostasis and adapting to stress stimuli (Debnath et al., 2023). In the context of GC development, autophagy acts as a “double-edged sword,” with its effects depending on the tumor microenvironment (TME) and genetic background. Among these, Autophagy-related Protein 1 (Ambra1), an important marker of the autophagic process (Li X. et al., 2022). It promotes the formation of class III PI3KC3 complexes by interacting with Beclin1 and Vps34/PI3KC3, thereby mediating the initial steps of autophagosome formation. In subsequent phases, autophagy-related genes (ATGs) such as ATG5, ATG7, ATG10, ATG12, and ATG16 drive autophagosomal membrane expansion, promoting autophagosome maturation and contributing to GC progression (Wang et al., 2021).

Cai T. et al. reported that AS-IV modulates autophagy through regulation of the Ambra1/Beclin1 complex, reducing both protein expression of Ambra1 and Beclin1 in tumor-bearing rat models and thereby exerting protective effects on gastric mucosa. Mechanistic analyses revealed that AS-IV significantly suppresses mRNA expression of ATG5, ATG12, and other core autophagy genes, providing direct evidence that autophagic gene networks are critical regulators of autophagic flux and GC progression (Cai et al., 2018). Notably, however, a potential caveat exists: excessive AS-IV exposure may paradoxically induce cytoprotective autophagy in tumor cells, enabling their survival via stress adaptation mechanisms. This risk may not only lead to the survival of tumor cells but also induce their proliferation. We believe that the “contradictory nature” of autophagy essentially stems from the adaptive response of its dynamic regulatory network to different physiological and pathological signals. This risk may not only lead to the survival of tumor cells but also induce proliferation. We believe that the “paradoxical” nature of autophagy is the adaptive response of its dynamic regulatory network to different physiological and pathological signals. This characteristic poses challenges for research and treatment. In the future, it is necessary to deeply analyze the key regulatory nodes of autophagy in specific environments to achieve precise intervention in the paradox of autophagy and promote its application in disease treatment.

### 4.4 Pyroptosis

Pyroptosis is an inflammatory form of programmed cell death mediated by inflammasome activation, characterized by plasma membrane pore formation, cell swelling and rupture, and the release of pro-inflammatory cytokines (such as Interleukin-1 beta [IL-1β]and Interleukin-18 [IL-18]) (Rao et al., 2022). It plays a critical role in pathological processes including infections, inflammatory diseases, and tumors. Emerging research in recent years has highlighted the substantial potential of AS-IV in leveraging pyroptotic mechanisms for tumor therapy (You et al., 2025). The canonical pyroptotic pathway involves regulation of caspase family proteins, enhancement of Caspase-1 expression or activity, and direct promotion of Gasdermin D (GSDMD) cleavage and inflammatory factor release (Makoni and Nichols, 2021).

Multiple studies have confirmed that AS-IV exerts dual mechanisms to induce pyroptosis. On one hand, it significantly increases Caspase-1 expression and activation, leading to GSDMD cleavage and initiation of pyroptosis (Li C. et al., 2023). On the other hand, it upregulates ROS levels to trigger and activate the NLRP3 inflammasome, which recruits and activates Caspase-1. This cascade results in the release of large quantities of mature IL-1β and IL-18, inducing inflammatory responses that drive pyroptotic cell death (Zhang et al., 2022; Fu and Wu, 2023). Collectively, these studies unravel the pyroptosis-inducing mechanisms of AS-IV and provide mechanistic validation for its role in suppressing GC progression via pyroptosis.

The pyroptosis and apoptosis of GC cells induced by AS-IV are centered on “inflammatory rupture” and “non-inflammatory autophagy” in mechanisms, respectively. The former relies on the caspase-1/GSDMD pathway accompanied by strong immune activation, while the latter achieves precise cell clearance through the caspase cascade reaction. The synergistic effect of the two may provide new strategies for the treatment of GC, but further studies on the selective regulatory mechanisms in different subtypes of GC (such as microsatellite instability type and HER2-positive type) are required to optimize clinical applications.

### 4.5 Angiogenesis

Tumor growth and metastatic dissemination are inherently dependent on neovascularization, a process by which nascent blood vessels supply tumor cells with essential nutrients, oxygen, and metastatic routes (Qian et al., 2016). Inhibiting tumor angiogenesis represents a key therapeutic mechanism of TCM in GC treatment, whereby TCM metabolites suppress tumor growth and metastasis by blocking angiogenesis-related factors, and depriving tumors of nutrient supply. AS-IV is a representative agent in this context.

Vascular endothelial growth factor (VEGF), a prototypical pro-angiogenic cytokine, exerts pleiotropic effects on vascular endothelial cells, including promotion of proliferation, migration, and survival, ultimately driving neovascularization (Khodabakhsh et al., 2021). Elevated VEGF expression in GC tissues correlates significantly with tumor progression, metastatic burden, and adverse clinical outcomes (Macedo et al., 2017). Experimental evidence demonstrates that AS-IV potently reduces intratumoral VEGF levels, concomitantly downregulating both protein and mRNA expression of matrix metalloproteinase-2 (MMP-2) and fibroblast growth factor 2 (FGF-2). These actions collectively impede tumor cell invasion, metastatic dissemination, and angiogenic sprouting (Zhang et al., 2017).

Notably, AS-IV has been shown to modulate both angiogenic and immune regulatory pathways. In a recent study, AS-IV enhanced antitumor immunity while suppressing angiogenesis by upregulating miR-195-5p and reducing programmed death-ligand 1 (PD-L1) expression in SGC7901 and MGC803 cell lines. This dual activity inhibits cell proliferation, epithelial-mesenchymal transition (EMT), and neovascularization (Liu et al., 2021). This study introduces a novel therapeutic strategy for GC: combination therapy with AS-IV and PD-1 inhibitors, offering promising insights for clinical translation.

### 4.6 TME

The immune system orchestrates a multifaceted role in tumor initiation, progression, and clinical outcome (Humphrey, 1971). Under physiological homeostasis, immunosurveillance mechanisms effectively eliminate transformed cells. But, chronic immune suppression permits cancer cells to evade detection, fostering uncontrolled proliferation and metastatic dissemination in tumor contexts (Ma et al., 2017). This immune evasion is intrinsically linked to dysregulation of the TME, a complex ecosystem characterized by dynamic crosstalk between fibroblasts and immune cell subsets (Anderson and Simon, 2020). Emerging evidence highlights bidirectional regulatory networks between fibroblasts and immune cells, which collectively govern tumor immunosurveillance and clearance (Di et al., 2024).

A defining feature of the TME is the “education” of normal fibroblasts into cancer-associated fibroblasts (CAFs), which exhibit striking heterogeneity and functional plasticity. As dominant stromal effectors, CAFs drive tumor progression by promoting cell proliferation, orchestrating immune escape pathways, inducing EMT, and mediating therapeutic resistance (Minoura et al., 2025; Song et al., 2025; Cao et al., 2025). Wang ZF et al. demonstrated that AS-IV induces microRNA reprogramming in gastric CAFs (GCAFs), specifically upregulating miR-214 and downregulating miR-301a expression in BGC-823-derived GCAFs. This epigenetic modulation abrogates pathological GCAF functions and reconfigures TME architecture (Wang et al., 2017). Concurrently, independent studies reveal that AS-IV suppresses the HOXA6/ZBTB12 transcriptional axis in GCAFs, reducing HOXA6 and ZBTB12 expression to attenuate GC cell malignancy and normalize TME composition (Liu et al., 2023). Collectively, these findings validate the TME as a critical therapeutic target in GC (Xiao and Yu, 2021). And they highlight AS-IV-mediated TME modulation as a promising new direction for GC therapy.

Based on the above discussions, we hypothesize that AS-IV regulates the TME through a network interaction of “anti-angiogenesis-programmed cell death-immune/stromal remodeling”. Its anti-angiogenic effect enhances the sensitivity of tumor cells to apoptosis/pyroptosis by improving the hypoxic microenvironment and reducing nutrient supply; in turn, factors released by programmed cell death (such as inflammatory factors and apoptotic bodies) affect angiogenesis and immune cell recruitment. This multi-target synergistic effect provides new therapeutic ideas for remodeling the TME, but preclinical studies are needed to clarify its selective regulatory mechanisms for different TME subtypes to achieve precise treatment. Meanwhile, various *in vivo* and *in vitro* experiments should continue to be conducted to corroborate this approach.

### 4.7 Ferroptosis

Ferroptosis is an oxidative cell death triggered by the excessive accumulation of iron-dependent lipid peroxides in the cell membrane, characterized by mitochondrial shrinkage, increased membrane density, and the absence of typical inflammatory responses. It is closely associated with glutathione (GSH) depletion and inhibition of glutathione peroxidase 4 (GPX4) activity, playing a critical role in pathological processes such as neurodegenerative diseases, cancer, and ischemia-reperfusion injury (Jiang et al., 2021).

Hepatic injury represents a common complication of cisplatin, a first-line chemotherapeutic agent for GC (Rha et al., 2023). Increasing evidence implicates ferroptosis—a non-apoptotic, iron-dependent form of lipid peroxidation-driven cell death—as a central executor of cisplatin-induced liver injury. Ferroptosis has therefore emerged as a promising strategy to mitigate chemotherapy-associated hepatotoxicity.

Guo J. et al. demonstrated that AS-IV potently alleviates cisplatin-induced hepatic injury, systemic inflammation, and oxidative stress. Mechanistically, AS-IV restores expression of glutathione peroxidase 4 (GPX4) and ferroptosis suppressor protein 1 (FSP1), two critical regulators of anti-ferroptotic defense, while significantly inhibiting iron-dependent lipid peroxidation and ferroptotic cell death (Guo et al., 2024). Correlative analyses revealed a strong positive correlation between ferroptotic activity and hepatic injury severity, such that enhanced lipid peroxidation correlates with exacerbated liver damage. These findings collectively establish AS-IV as an effective protector against cisplatin hepatotoxicity through ferroptosis inhibition. Meanwhile, they highlight its translational potential in managing ferroptosis-mediated complications of GC treatment. Some scholars believe that AS-IV’s inhibition of ferroptosis has certain interactive relationships with other forms of programmed cell death, such as synergism with apoptosis and antagonism with pyroptosis. However, the limitation is that these hypotheses all require further experimental verification.

### 4.8 Inflammation

Inflammation is intricately woven into the entire spectrum of GC initiation and progression, forming a complex bidirectional relationship characterized by mutual promotion (Jaroenlapnopparat et al., 2022). On one hand, chronic inflammation serves as a key initiator of gastric carcinogenesis. Through activation of canonical signaling pathways such as NF-κB and JAK-STAT, chronic inflammation induces gastric mucosal injury, aberrant cell proliferation, and premalignant lesions, ultimately culminating in GC. On the other hand, inflammatory responses within the TME act as accomplices in cancer progression. Pro-tumorigenic inflammatory cells release cytokines such as IL-6 and TNF-α, which promote tumor cell proliferation, invasion, and immune evasion while exacerbating chemoresistance and immunotherapy resistance.

More and more studies have demonstrated significant anti-inflammatory properties of AS-IV (Liang et al., 2023). The NF-κB pathway, a classical regulator of inflammation, represents a central target of AS-IV’s anti-inflammatory effects. A meta-analysis has shown that AS-IV potently suppresses the release of pro-inflammatory cytokines (TNF-α, IL-1β, IL-6) mediated by the NF-κB signaling axis, thereby alleviating tissue structural and functional damage (Li L. et al., 2023). These findings underscore the therapeutic potential of targeting inflammatory pathways with AS-IV for both gastritis management and primary prevention of GC, bridging the gap between anti-inflammatory pharmacology and oncology prevention.

### 4.9 Helicobacter pylori

As previously mentioned, *Helicobacter pylori* is a major cause of GC. Eradication of *H. pylori* can effectively reduce the incidence of GC and GC-related mortality, particularly in East Asian countries (Ford et al., 2020). The potential advantage of AS-IV lies in its multi-target inhibition of *H. pylori* infection and reduction of GC risk. It can regulate the host immune-inflammatory response, reduce the release of pro-inflammatory cytokines, and alleviate inflammatory damage to the gastric mucosa. By indirectly inhibiting the survival microenvironment of *H. pylori*, it effectively blocks the pathological process of “infection-inflammation-carcinogenesis”. Chronic atrophic gastritis (CAG) is a chronic inflammatory condition triggered by *H. pylori* infection. Its persistent progression can evolve into GC through pathological processes such as intestinal metaplasia and dysplasia, serving as a critical intermediate link between *H. pylori* infection and GC (Tran et al., 2024).

Duan B et al. demonstrated through *in vivo* and *in vitro* experiments that AS-IV exerts protective effects on the gastric mucosa in CAG rats by activating the thrombin/Protease-Activated Receptor 1 (PAR-1) signaling pathway, inhibiting NF-κB p65 phosphorylation, and reducing the expression of cyclooxygenase-2 (COX-2) and inflammatory cytokines (TNF-α, IL-6, etc.), thereby alleviating gastric mucosal injury (Duan et al., 2025). This study provides a theoretical basis for the application of AS-IV in the treatment of CAG, yet the specific action targets and long-term efficacy still require further investigation. Additionally, another study has shown that TCM formulas containing AS-IV can effectively increase the proliferation rate of GES-1 cells, inhibit apoptosis, and exhibit significant anti- *H. pylori* activity (Hu et al., 2022). However, current related research is mostly limited to *in vitro* cell experiments or animal models, lacking support from large-sample clinical data. The specific action targets, effective dosage, and long-term intervention effects in humans remain unclear, urgently requiring more high-quality studies to reveal its complete mechanism.

## 5 Conclusion and recommendations

**FIGURE 3:**
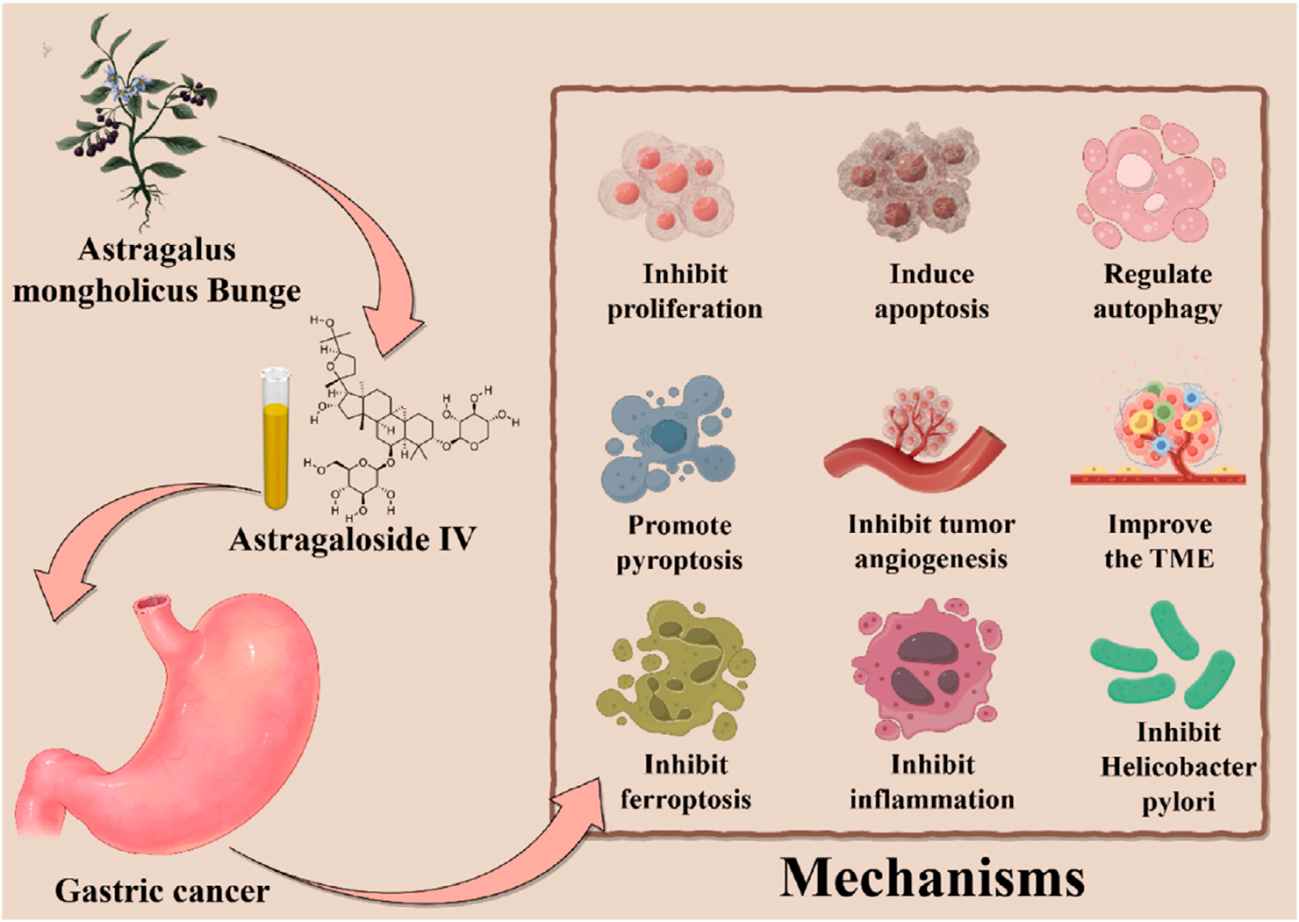
Mechanisms of Astragaloside IV in the treatment of GC.

In summary, this review systematically elucidates the multi-faceted mechanisms underlying AS-IV in GC therapy. And it demonstrates AS-IV is a potential nemesis for GC. On one hand, a defining feature of AS-IV is its ability to induce multiple forms of programmed cell death—including apoptosis, pyroptosis, autophagy, and ferroptosis—in a multi-targeted manner, representing a major therapeutic highlight. On the other hand, AS-IV delays GC progression through parallel mechanisms: inhibiting cellular proliferation, blocking tumor angiogenesis and metastasis, reprogramming the TME, inhibiting *H. pylori* and suppressing chronic inflammation. These multi-pronged effects collectively establish AS-IV as a promising candidate for GC treatment, opening new avenues for therapeutic development (Table 1; Figure 3).

**TABLE 1:** Experiments mainly on the mechanisms of AS‐IV in the treatment of GC.

Pharmacological effects	Types	Models	Concentration	Treatment duration	Control groups	Mechanisms	References
Inhibit proliferation	*In vitro*	GC cells (HGC-27 and MKN-45)	0,10,20,40 μg/mL	—	GES cells	Down-regulating the expression of circDLST relieves the adsorption of miR-489-3p, targets and inhibits the expression of EIF4A1, thereby suppressing the proliferation, migration and invasion of GC cells	Li F. et al. (2022)
	*In vivo*	Tumor-bearing nude mice	40 mg/kg	21 days	1.Control group + vector 2.AS-IV + vector 3.AS-IV + circDLST	Down-regulating the expression of circDLST in tumor tissues activates the miR-489-3p/EIF4A1 pathway, thereby inhibiting the growth of GC tumors in nude mice	
Induce apoptosis	*In vitro*	SW480 cells, HepG2 cells, etc.	5–20 μM	—	1.Blank control group 2.Model group 3.Drug (positive) control group	1.The membrane potential decreases and permeability increases, activating Caspase 3, Caspase-7 and Caspase-9, and producing cleavage activation 2.Upregulate the expression of Bax and inhibite the expression of Bcl-2 increases the Bax/Bcl-2 ratio, thereby promoting changes in mitochondrial membrane permeability	Xia et al. (2023), Eskandari and Eaves (2022), Campbell and Tait (2018), Zhao et al. (2019)
Regulate autophagy	*In vivo*	Rats with gastric precancerous lesions (GPL)	50,100 mg/kg	10 weeks	1.Blank control group 2.AS-IV low-dose group 3.AS-IV high-dose group	Down-regulating the expression of p53 inhibits the formation of the Ambra1/Beclin1 complex, thereby reducing the expression of autophagy-related proteins (such as ATG5, ATG12, and p62), and ultimately suppressing the excessive autophagy of gastric mucosal cells in GPL rats	Cai et al. (2018)
Promote pyroptosis	*In vitro*	Bone marrow-derived macrophages (BMDMs)	100 μM	—	1.Blank control group 2.Model group 3.AS-IV group	Regulate macrophage function through the ROS/NLRP3/Caspase-1/GSDMD axis to trigger pyroptosis	Li C. et al. (2023), Zhang et al. (2022), Fu and Wu (2023)
Inhibit tumor angiogenesis	*In vivo*	Tumor-bearing nude mice	20 mg/kg	21 days	1.Blank control group 2.Cisplatin group 3.AS-IV group 4.Curcumin group 5.Combination group	Reduce the number of tumor microvessels effectively and downregulate the expression of pro-angiogenic factors such as VEGF, FGF2, and MMP2	Zhang et al. (2017)
	*In vitro*	SGC7901 and MGC803 cells	10 μg/mL	—	1.Blank control group 2.Solvent control group 3.Negative control group 4.Model control group	Up-regulating miR-195-5p inhibits the expression of its target PD-L1, thereby suppressing EMT and angiogenesis in gastric cancer cells and exerting anti-tumor effects	Liu et al. (2021)
Improve the TME	*In vitro*	GNFs, GCAFs,BGC-823 cells	10,20,40 μmol/L	—	1.Blank control group 2.Model group 3.Solvent control group 4.Negative control group 5.Combined treatment control group	1.Up-regulating the expression of miR-214 and down-regulating miR-301a occurs in GCAFs 2.Regulate the HOXA6/ZBTB12 axis to inhibit the expression levels of HOXA6 and ZBTB12 in GCAFs	Wang et al. (2017), Liu et al. (2023)
Inhibit ferroptosis	*In vivo*	Cisplatin-induced liver injury model in mice	40,80 mg/kg	9 days	1.Blank control group 2.Low-dose group 3.High-dose group	Restore the expression of GPX4 and FSP1, and potently inhibit ferroptosis	Guo et al. (2024)
Inhibit Inflammation	*In vitro*	HepG2 cells, HK-2 cells, etc.	12.5 μM-200 μg/mL	—	Model group	Inhibit the release of inflammatory factors such as TNF-α, IL-1β and IL-6, and reduce tissue structural and functional damage	Li et al. (2023)
Inhibit *Helicobacter pylori*	*In vitro*	GES-1 human normal gastric epithelial cells	20 μmol/L	—	1.Blank control group 2.Model group 3.Inhibitor control group	Up-regulating the PAR-1 expression can inhibit the NF-κB p65/COX-2 inflammatory axis, thereby reducing the inflammatory injury and apoptosis of gastric epithelial cells induced by MNNG.	Duan et al. (2025)
	*In vivo*	Sprague-Dawley rat	50,100 mg/kg	10 weeks	1.Blank control group 2.CAG model group 3.AS-IV low-dose group 4.AS-IV high-dose group	Up-regulating the PAR-1 expression inhibits the activation of NF-κB p65 and COX-2, reduces the release of inflammatory factors, and thus alleviates MNNG-induced gastric mucosal injury	
	*In vitro*	GES-1 human normal gastric epithelial cells	2.08,4.16 mg/mL	—	1.Blank control group 2.*H. pylori* infection model group 3.Low-dose group 4.High-dose group 5.Amoxicillin positive control group	Significantly improve the survival rate of GES-1 cells infected with *H. pylori,* reduce the cell apoptosis rate, and decrease the mRNA expression of virulence factors such as HpPrtC, OPiA, IceA1, and BabA2. The effect is dose-dependent, thereby inhibiting the activity of *H. pylori*	Hu et al. (2022)

Although significant advancements have been made in the research on AS-IV for GC treatment, several crucial limitations and challenges remain. For instance.

### 5.1 Overreliance on preclinical models

Despite significant progress, the majority of AS-IV studies in GC remain preclinical, with limited clinical translation (Chen et al., 2021). Many investigations rely heavily on *in vitro* models, which fail to recapitulate the complexity of the TME or predict human pharmacodynamics. To address this, interdisciplinary approaches integrating *in vitro*/*in vivo* models and clinical trials are urgently needed to provide robust mechanistic validation and therapeutic efficacy data.

### 5.2 Unclear toxicological profile

Although various studies have reported the therapeutic doses of AS-IV, there is relatively little attention paid to its toxicity. Existing studies have shown that AS-IV has no obvious toxicity or adverse reactions. For example, Xuying W et al. intravenously injected AS-IV into SD rats at doses of 0.25, 0.5, and 1.0 mg/kg per day before mating for 4 consecutive weeks, continuing until the 6th day of pregnancy in females, to evaluate the fertility and early embryonic developmental toxicity of the rats. In addition, the perinatal toxicity of the rats was evaluated from gestational day (GD) 15–21 and lactation day (LD) 1–21. The results showed that AS-IV did not detect toxicity in the maternal body of rats in the range of 0.25–1.0 mg/kg. Further results showed that compared with the control group, maternal exposure to a dose of 1.0 mg/kg per day would have a certain inhibitory effect on the fur development of the pups, but it would not affect the memory and learning of the pups (Xuying et al., 2010). This study systematically evaluated the acute toxicity, maternal toxicity, embryotoxicity, and fetotoxicity of AS-IV, proving its safety. However, it must be pointed out that there are few clinical studies on AS-IV, and there is a lack of long-term toxicity data, long-term clinical outcome tracking, and follow-up. Especially now that the combination of AS-IV with chemotherapy and immunotherapy for GC has become a popular trend. But whether this model will produce toxic and side effects still requires more experiments to prove its safety.

### 5.3 Suboptimal bioavailability

Oral administration of AS-IV is hindered by its low absolute bioavailability (7.4%), attributed to poor intestinal permeability, high molecular weight, and low lipophilicity (Zhang et al., 2007). Innovative strategies are required to enhance systemic exposure: First, Nanotechnology-based Drug Delivery. Development of novel formulations, such as nanoparticles or hydrogels, can improve bioavailability and enable targeted delivery. The unique physicochemical properties of AS-IV provide a theoretical basis for the development of nano-formulations for GC biological barriers. The amphiphilic structure of AS-IV endows it with a balance of water solubility and lipid solubility, which helps to achieve solubilization and targeted delivery through nanocarriers. Its stable glycosidic bond structure exhibits good chemical stability in physiological environments, ensuring the integrity of nano-formulations during *in vivo* circulation. Based on the surface activity and intermolecular forces of AS-IV, technologies such as nanoprecipitation and emulsion solvent evaporation can be used to precisely regulate the particle size, surface charge, and morphology of nanoparticles, enabling them to effectively penetrate the physiological barriers of GC tissues. Meanwhile, leveraging its potential bioadhesion and specific interactions with the TME, a nano-delivery system with both active targeting and barrier regulation functions can be constructed to achieve efficient enrichment and sustained release of drugs at the site of GC. For example, Li X. et al. designed a pH-responsive hydrogel incorporating AS-IV-loaded nanoparticles to sustain drug release and induce ferroptosis in tumor cells (Li X. et al., 2025). Second, Gut Microbiome Modulation. Leveraging gut lactobacilli to enhance AS-IV metabolism represents a promising avenue, as intestinal microbiota play a critical role in its absorption and activation.

Finally, with ongoing mechanistic elucidation and technological innovation, AS-IV holds substantial promise for GC therapy. Addressing current limitations through multidisciplinary collaboration—including pharmacokinetics, nanomedicine, and clinical oncology—will accelerate its translation into evidence-based clinical applications, offering new hope for patients with this devastating disease.
